# Is Curcumin at the Threshold of Therapeutic Effectiveness on Patients with Colon Cancer?—A Systematic Review

**DOI:** 10.3389/fphar.2021.707231

**Published:** 2021-09-02

**Authors:** Laila Khaled Ismael Abdelgawad Shafei, Mohamed Izham Mohamed Ibrahim, Nashiru Billa

**Affiliations:** ^1^ College of Pharmacy, QU Health, Qatar University, Doha, Qatar; ^2^ Department of Clinical of Pharmacy and Practice, College of Pharmacy, QU Health Qatar University, Doha, Qatar; ^3^ Department of Pharmaceutical Sciences, College of Pharmacy, QU Health Qatar University, Doha, Qatar

**Keywords:** curcumin, colon, cancer, clinical trial, systematic review, outcome measures, formulation, nanoparticle delivery

## Abstract

Curcumin, obtained from curcuma longa, has been the subject of decades of scientific investigation on its therapeutic usefulness. It is reported to possess several therapeutic properties, of which anti-colon cancer is of interest in this review. Clinically however, curcumin has yet to firm up its place among established anti-colon cancer therapeutic contenders. We aimed to systematically review prevailing clinical evidence on the role of curcumin in colon cancer treatment. The review drawing from literature on clinical studies indicates fairly long term tolerability. No regression of tumor was reported when curcumin was the sole intervention. Increase in p53 level expression was reported in a placebo controlled study but no reduction in PGE2 or 5HETE. Pharmacokinetic data on healthy humans indicate that formulated curcumin delivery systems present significantly higher systemic bioavailability. It appears therefore that the clinical use of curcumin can potentially be realized only through appropriate formulation interventions.

**Systematic Review Registration**: [website], identifier [registration number]

## Introduction

Colon cancer is presented initially, as polyps within the colon epithelia and gradually transforms into cancerous tissue (Simon, 2016). Diet, age and genetic predisposition have been recognised as contributing factors to triggering colon cancer. Even though this disease is currently recognized as the third most common cause of deaths amongst cancer patients (Bray et al., 2018), rates from colon cancer patients have been dropping over several decades (Bray et al., 2018), thanks to advances in diagnostic technologies such as endoscopy, which can be used for early detection of polyps during screening. Notwithstanding, colon cancer remains existentially formidable clinical condition and reliance on early detection alone is insufficient to address the extent of mortality because most cases are presented only after transformation into advanced states.

Conventionally, colon cancer treatments include tissue resection, radiation therapy or more commonly, chemotherapy, which is also the least invasive and least costly and most convenient administer in the management of the disease. On the other hand, chemotherapy is associated with severe systemic side effects, sometimes resulting in life-threatening or fatal consequences. Thus, there is growing interests in the use of safer chemotherapeutics or application of targeted formulation technologies, aimed at mitigating the side effects that arise from use of chemotherapeutics. Furthermore, some chemotherapeutics directly derived from plant origin have been found to present effective anticancer effects at equi-molar concentrations to commonly used chemotherapeutics and yet present relatively fewer side effects (Law et al., 2020). One such anticancer agent which has received wide attention by researchers over the past decades is curcumin. It is one of the key constituents found in the Indian spice turmeric (curcuma longa), with anti-diabetic, anti-fungal, anti-oxidant, anti-inflammatory, anti-HIV, anti-angiogenic, anti-mutagenic, and anti-cancer properties (Vareed et al., 2008; Nelson et al., 2017; Wiggers et al., 2017).

Aptly, the incidence of colon cancer in the Indian subcontinent is among the lowest of colon cancer (Pathy et al., 2012), possibly due to a regular intake of the spice as it is a key constituent in their diet. Therefore, it is very likely that some form of chemoprevention is offered by dietary consumption of turmeric. This notion has prompted researchers to further explore the full potential of curcumin in not just colon cancer treatment but other cancers as well through (i) formulation approaches, including some from our own labs (Chuah et al., 2014; Sabra et al., 2019), (ii) chemical synthetic approaches Chaubey et al. (2018), Sabra et al. (2019), Law et al. (2020) and (iii) formulation combined with chemical synthetic approaches (Jin et al., 2009; Li et al., 2014; Wong et al., 2019). Curcumin has multiple proposed mechanisms of actions on colon cancer; for example, it modulates Wnt/β-catenin pathway whereby dysregulation of this pathway leads to accumulation of β-catenin, which enhances the expression of multiple oncogenes. (Li et al., 2014; Wang et al., 2019). In addition, curcumin affects the PI3K/Akt and because this misregulation of this gene is correlated with the carcinogenesis process, inhibition of PI3K/Akt augmented by curcumin, induces cell death and apoptosis (Wang et al., 2019). Moreover, curcumin is believed to affect JAK/STAT, MAPK, p53, and NF-ĸb pathways which are considered common signalling pathways in the pathophysiology of cancer (Wang et al., 2019). The consequences of these inhibitions by curcumin is still a subject of investigation and will require further clinical trials to fully present the mechanism (Vareed et al., 2008). Furthermore, curcumin also orchestrates the expression of oncogenic and tumor-suppressive miRNAs, whereby, when miRNAs are inhibited, there is a resultant inhibition of carcinogenesis and promotion of apoptosis of cancerous cells (Wang et al., 2019). Generally, at supra-therapeutic doses, chemotherapeutic agents present toxicities. We dedicate a significant portion of this review to presenting reported toxicities from curcumin administration in clinical trials. Another motivation to the utilization of curcumin in colon cancer treatment is the possible tolerability by patients. In spite of the extensive research aimed at realizing the use of curcumin for colon cancer treatment, there has yet to be a viable formulation on the market, or that curcumin has yet to be clinically recognized with worldwide acclaim in treating colon cancer. This lack of traction in the lead to the materialization of clinical use of curcumin is party borne of the fact that it is poorly soluble and unstable, both contributing to the constraints limiting the deployment of curcumin in colon cancer therapy (Sabra et al., 2019). It is thus not surprising that most of the research aimed at establishing the anti-colon cancer potential of curcumin have been at the *in vitro* level, with only a handful transcending the clinical trial threshold. On the other hand, there are currently about 150 clinical trials registered on use of curcumin for various clinical conditions, including cancer, attesting to its safety profile (Wong et al., 2019). Therefore, it is relevant to assess the trajectory of curcumin utilization towards materialization as a prospective, effective and/or superior anticancer agent when compared to more commonly used anticancer agents. There is no evidence of a systematic review conducted on the effectiveness of curcumin in patients with colon cancer. Thus, this systematic review aims to shed light on current trends and explore the prospects of clinical application of curcumin in managing colon cancer. Secondarily, we aim to provide a framework for the safety profile in use of curcumin on colon cancer patients.

## Methods

The study design was based on a systematic review of the current primary literature and clinical trials on the effectiveness of curcumin in patients with colon cancer. The review was conducted according to the Preferred Reporting Items for Systematic Reviews and Meta-Analyses (PRISMA) protocol.

### Search Strategy

The literature search for relevant articles was conducted systematically *via* three databases: PUBMED, MEDLINE, and SCOPUS. Other relevant articles not included in the above databases were manually extracted from google scholar after scanning the reference list from relevant articles pooled from the databases. The search terms generated were based on the population, intervention, comparison, outcome (PICO) question: “What is the effectiveness of curcumin on patients with colon cancer?” Our search strategy for the population was based on the domain “colon cancer” (e.g., “Colonic neoplasms” Or “Colon Cancer”), whilst for the intervention or investigated agent curcumin, the terms used were (e.g., Curcumin or Turmeric or Curcuma Longa). The comparator Terms included “Antineoplastic Agents” or “Anticancer Agents’. The outcome mainly focused on any effects that resulted from the clinical trial. The main search domain was connected using the Boolean operator “AND”, whilst each term within the domain was connected by the database in combination with the database-specific filters such as original text, English language, full text, Mesh filters, and exclusion of the results where applicable, Table 1.

**TABLE 1:** Search terms generated in each database

PubMed	((“Colonic Neoplasms”[Mesh]) AND (Curcumin OR Turmeric OR curcuma longa)) AND (“Antineoplastic Agents”[Mesh])
Medline	(Colonic Neoplasms OR Colon cancer) AND (Curcumin OR Turmeric OR curcuma longa) AND (Antineoplastic Agents)
SOCPUS	[TITLE-ABS-KEY (“Colonic Neoplasms” OR “Colon cancer”)] AND [TITLE-ABS-KEY (curcumin OR turmeric OR “curcuma longa”)] AND [TITLE-ABS-KEY (“Antineoplastic Agents" OR "Anti-Cancer Agents" )]

### Selection Criteria

The literature search involved adjusting certain eligibility criteria in order to match the remit of the present systematic review. Eligibility for article selection included English language as the medium of publication, full-texted, and involving clinical trials on human subjects with colon cancer. Patients with other types of cancers were not included in the review. The durational scope of the data retrieved was between the year 2000 till 2021. Furthermore, all *in vitro* and animals were not included in the review.

### Data Collection and Analyses

The data included in the review were extracted using a predesigned data extraction tool, where the output was collated into (i) general information about the trials, (ii) form in which curcumin was administered (iii) methods and outcomes from each trial. The data also included the number of patients in the intervention and comparator, stage of colon cancer, intervention, regression of cancer, reported adverse drug reactions, and study conclusions. Extracted data were then analyzed qualitatively and reported subjectively and descriptively in the results section using the PRISMA protocol.

### Quality Assessment

The clinical trials included in the review were both randomized control trials (RCTs), and quasi-experimental. In some studies, patients presented with metastatic colon cancer without a comparator arm. The quality assessment of the collated studies was conducted by two researchers and any major discrepancies between their analyses were resolved by a third researcher. The quality check was completed using the Joanna Briggs Institute (JBI) critical appraisal checklist (Critical Appraisal tools, 2021). This checklist provides for quality of both randomized control trials and non-randomized control trials (quasi-experimental studies) (Ma et al., 2020). A 13-item questionnaire was used to assess the randomization, allocation, blinding, results, and the statistical methods used in the RCTs. On the other hand, the JBI checklist used for quasi-experimental studies included nine items that assesses interventions and outcomes. The appraisal was defined as to whether to include, exclude, or seek further information.

### Statistical Analyses

Data input and statistical analyses were performed using Excel program. Descriptive statistics for the quality scores of the included articles were calculated and the intraclass correlation was calculated between the results of the two assessors.

## Results

### Literature Search

**FIGURE 1:**
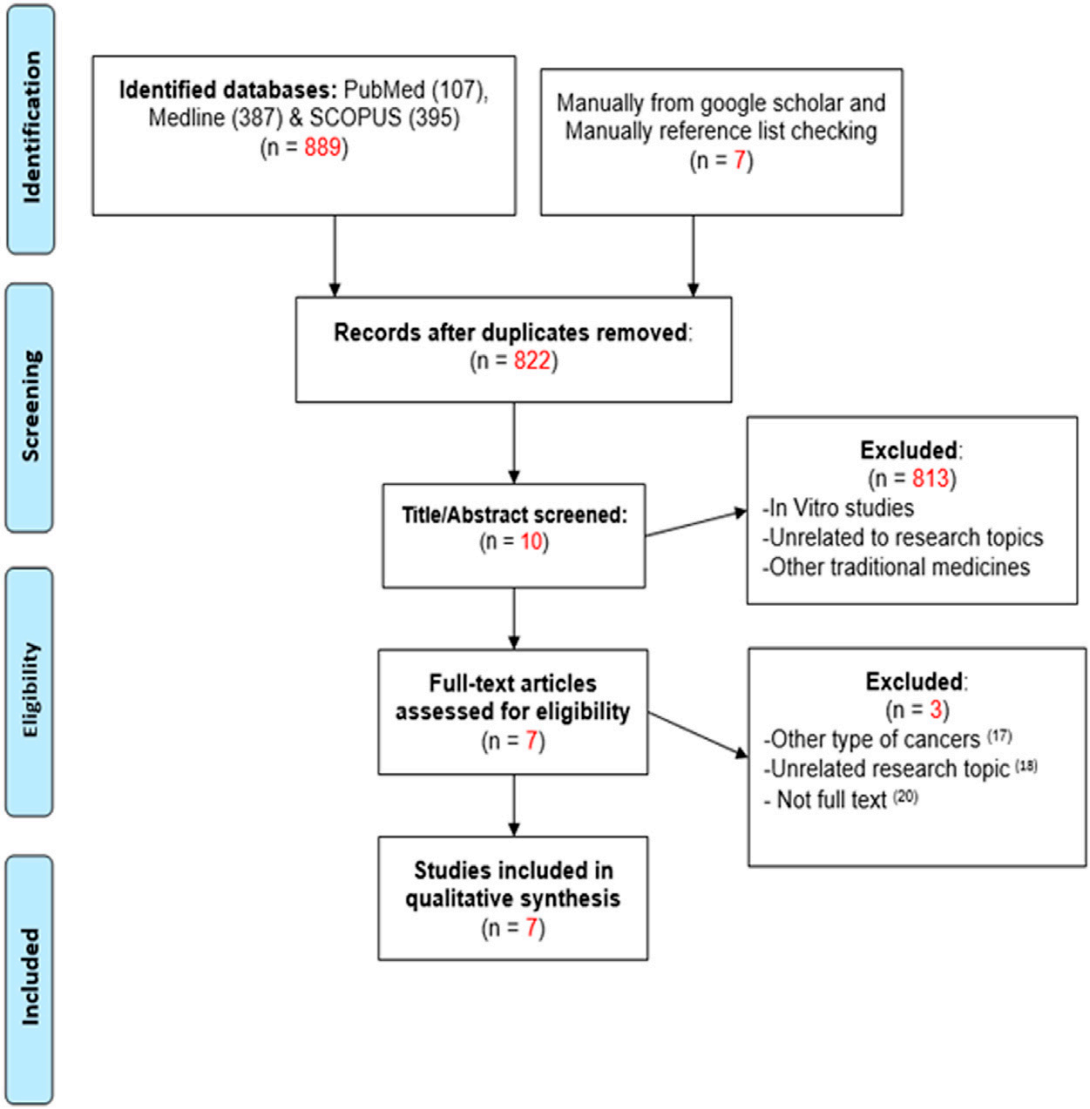
The PRISMA flow diagram for article selection.

A total of 896 articles were identified and collated from all sources: PUBMED, MEDLINE, SCOPUS, and the grey literature. After data cleaning for duplicates, 822 articles were pooled. As shown in the PRISMA flow diagram (Figure 1), the total number of articles that met the eligibility criteria was 10, after which, full-text screening yielded seven eligible articles. Of these, three were RCT and 4, quasi-experimental studies. Three articles were excluded by full-text screening, because one of the studies was conducted on pancreatic cancer patients whilst the other study was aimed at assessing the pharmacokinetic profile of curcumin, both of which are out of the scope of this systematic review (He et al., 2011; Kanai et al., 2013; Allegra et al., 2017). The third study was a protocol, which was also not within the scope of the present systematic review (Irving et al., 2015). Seven articles were included in the qualitative analysis because they matched the inclusion criteria of this review (Hsieh, 2001; Sharma et al., 2001; Carroll et al., 2011; He et al., 2011; Fang et al., 2014; Greil et al., 2018; Howells et al., 2019).

### Studies Characteristics

As shown in Table 2, two articles were published in 2001 (Hsieh, 2001; Sharma et al., 2001) and 1 in 2004 (Carroll et al., 2011), whilst the remaining 5 between 2011 and 2019.Three articles were published in United Kingdom (Sharma et al., 2001; Carroll et al., 2011; Howells et al., 2019), two from China (Hsieh, 2001; He et al., 2011), one each from Australia and the United States. The study objectives were common to all studies, i.e., to assess the efficacy of curcumin on patients with colon cancer. Only three studies were designated as a comparative study between curcumin and other interventions (He et al., 2011; Fang et al., 2014; Greil et al., 2018). The remaining studies were designated as single intervention with curcumin only (Hsieh, 2001; Sharma et al., 2001; Carroll et al., 2011; Howells et al., 2019). As indicated earlier, clinical studies focused on patients with colon cancer and no other cancers, as shown in Table 2. Table 3 presents the dosage form administered, dose and duration.

**TABLE 2:** Study objectives of included articles.

Article	Year	Country/Region	Study design	Study objective
(20)	2018	Australia/Oceania	Quasi-experimental	Safety and tolerability of increased doses of liposomal curcumin in patients with metastatic cancer.
(21)	2019	United Kingdom/Europe	RCT	Safety, efficacy, quality of life, neurotoxicity of curcuminoids and C-X-C-motif chemokine ligand 1 in patients receiving FOLFOX compared with FOLFOX + 2 g oral curcumin/d (CUFOX).
(22)	2001	United Kingdom/Europe	Quasi-experimental	Safety of p.o curcumin administered as extracts.
(23)	2001	China/Asia	Quasi-experimental	A phase 1 study on toxicity and chemoprevention of curcumin in high risk cancer patients.
(24)	2004	United Kingdom/Europe	Quasi-experimental	Toxicity of high curcumin doses administered orally to patients with advanced cancer.
(25)	2011	China/Asia	RCT	Inhibitory mechanism of curcumin on cancer cells from patients with colorectal cancer
(26)	2011	United States/North America	RCT	Effect of curcumin on PGE2 and assessment of tolerability in colon cancer patients with pre-neoplasms.

**TABLE 3:** curcumin regimen in studied articles.

Article	Formulation	Dose	Route	Frequency	Duration
20	Liposomal curcumin formulation	100 mg/ ml for 8 hr 300 mg/ ml for 6 hr or 8 hr	IV infusion	Once weekly	8 weeks
21	Curcumin C3 complex/d (Sabinsa Corp—containing ∼80% Curcumin)+ FOLFOX + bevacizumab called “CUFOX”	2000 mg	Oral	Every 2 weeks for ≤ 12 cycles	Until patient progression, unacceptable toxicity, death, or withdrawn consent
22	Curcuminoids extract suspension in Curcuma Spp*.* essential oils	440, 880, 1320, 1760, and 2200 mg	Oral	Daily	Until disease progression orwithdrawn consent
23	Curcumin tablets	500 mg	Oral	Daily	Three months or until signs of toxicity developed
24	Curcuminoids in hard gelatine capsules	500 mg	Oral	Daily	Until disease progression orwithdrawn consent
25	Curcumin in hard gelatine capsule	360 mg	Oral	Daily	10-30 days pre-surgery to differentcohort of patients
26	Curcumin in micronized powder	curcumin at 2000 mg initially, then 4000 mg	Oral	Daily	Until biopsy conducted

### Quality Assessment

The seven collated studies included three RCT He et al. (2011), Fang et al. (2014), Howells et al. (2019), and four were quasi experimental studies (Hsieh, 2001; Sharma et al., 2001; Carroll et al., 2011; Greil et al., 2018). Four studies were classified as high quality (3 RCTs and 1 Quasi-experimental study), whilst three studies were moderate quality (all quasi-experimental studies) Table 4 **.**

**TABLE 4:** Outcomes and conclusions.

Article	Stage of colon cancer	Intervention	Comparator	Regression of cancer	Adverse drug reactions	Study conclusion
20	Metastatic Cancer	Curcumin	None	After 8 weeks 23 patients showed progressive disease	Haematological ADRs; facial enema, anaemia, echinocytes and red blood cell abnormalities	Dose of 300 mg/ ml liposomal curcumin monotherapy over 6 h is warranted as the recommended starting dose for future anti-cancer clinical trials.
21	MetastaticCancer	FOLFOX ± bevacizumab plus curcumin(CUFOX)	FOLFOX ± bevacizumab	Out of 22, 18 patients died on CUFOX. 2 on CUFOX proceeded to surgical resection of liver metastases. No complete responses were observed	Abdominal pain, acute kidney injury, anorexia, bloating, constipation, diarrhoea, dry mouth, dyspepsia, flatulence, nausea, oral mucositis and vomiting	Combination of curcumin with FOLFOX chemotherapy represents a safe and tolerable treatment with potential to provide patient benefit.
22	Adenocarcinoma(No Specified stage)	Curcuminoids	None	5 patients exhibited stable disease on CT scan: 3 patients on 440, 880, and 1760 mg of curcuma extract for 3 months; 2 patients on 880 and 1320 mg of Curcuma extract for 4 months.	Generally, well tolerated at all dose levels.Nausea and Diarrhoea	Doses of up to 2200 mg of curcuma extract (containing 180 mg of curcumin) per day can be administered to patients with cancer for up to 4 months, focusing on the effects of such doses on target tissues, particularly colon epithelium.
23	Premalignant lesions in colon	Curcumin	None	Histological improvement of the precancerous lesions observed in 7 out of 25 patients with various high-risk and pre-malignant lesions	No ADRs observed up to level 5 (8000 mg/ day)	Recommended oral dose of curcumin for future phase II studies is 6000 - 8000 mg/ day.
24	Adenocarcinoma(No Specified stage)	Curcumin (previously on chemotherapy)	None	Decreases in tumour markers or serum cholesterol not attributable to treatment in any of the patients	Diarrhoea and nausea	Daily dose of 3600 mg of curcumin suitable for evaluation in the prevention of malignancies at sites other than the gastrointestinal tract.
25	No Specified Stage	Curcumin + chemotherapy or curcumin + chemotherapy + radiotherapy	Placebo	Increases the p53 expression.	Diarrhoea in both groups during the study period; 8 and 10 patients, respectively.	Curcumin administration can be a supplemental to remedy in colon cancer treatment.
26	High risk of colon cancer	Curcumin	None	No reduction in PGE2 or 5-HETE in ACF	25 of 41 participants had grade-1 and -2 toxicities, primarily gastrointestinal disturbances, including diarrhoea	Pure curcumin significantly reduced ACF number in humans, confirming preclinical observations regarding changes in ACF in response to curcuminoid mixtures.

The results of the quality assessment by the JBI tool are presented in Table 5, where the questionnaire of the tool was answered as “yes”, “no” or “unclear”; then the percentage of questions answered as “yes” was calculated, to estimate the quality of the article. Questions 10–13 were answered as not applicable because these items do not apply to articles that were quasi-experimental studies. As per the results, only three articles had the highest quality with a 77% of “yes” response (Sharma et al., 2001; He et al., 2011; Fang et al., 2014). However, the remaining articles were of moderate quality (Hsieh, 2001; Carroll et al., 2011; Greil et al., 2018), and only one study showed the lowest quality with 53% response (Howells et al., 2019). An interclass correlation was carried out to assess the agreement between the two assessors who performed the quality assessment, the correlation ranged between 0.118 and 0.861. The correlation between assessors for all studies we were 0.5 and above (Hsieh, 2001; Sharma et al., 2001; Carroll et al., 2011; Fang et al., 2014; Greil et al., 2018; Howells et al., 2019); however, only one study showed the least correlation (He et al., 2011).

**TABLE 5:** Critical appraisal of eligible studies.

Article	Q1	Q2	Q3	Q4	Q5	Q6	Q7	Q8	Q9	Q10	Q11	Q12	Q13	% yes response	Intraclass correlation scores
20	Y	N	U	U	Y	Y	U	Y	Y	NA	NA	NA	NA	55%	0.739
21	Y	Y	N	N	N	Y	Y	U	U	Y	Y	Y	U	53%	0.613
22	Y	Y	U	U	Y	Y	Y	Y	Y	NA	NA	NA	NA	77%	0.861
23	Y	U	N	N	Y	Y	Y	Y	Y	NA	NA	NA	NA	66%	0.546
24	Y	Y	U	U	Y	U	Y	U	Y	NA	NA	NA	NA	55%	0.783
25	Y	Y	Y	Y	U	U	Y	Y	Y	Y	Y	U	Y	77%	0.118
26	Y	Y	Y	Y	Y	U	U	Y	N	Y	Y	Y	Y	77%	0.613

In most of the trials, intervention was based on oral administration of curcumin, except in one study where patients were administered curcumin by IV infusion (Greil et al., 2018). Oral doses ranged between 2 and 6 g of curcumin, with 500 mg administered in two studies (Hsieh, 2001; Carroll et al., 2011), and 360 mg in another (He et al., 2011), while in three studies, 2 g of curcumin was used (Sharma et al., 2001; Fang et al., 2014; Howells et al., 2019).

As illustrated in Table 4, the effectiveness of curcumin was somewhat inconclusive, with only two studies indicating that curcumin was effective in molecular and histological levels (Hsieh, 2001; He et al., 2011). In the other studies, the disease had progressed (Carroll et al., 2011; Greil et al., 2018), whilst in another, a fatality was reported, with two patients developing liver metastasis (Howells et al., 2019). Most of the studies reported the same adverse drug reactions to curcumin, which included nausea and diarrhea (Sharma et al., 2001; Carroll et al., 2011; He et al., 2011; Fang et al., 2014; Howells et al., 2019). In 2 studies where curcumin was administered as IV and in the other trial where curcumin was administered along with FOLFOX, other hematological side effects, anemia and anorexia were reported (Greil et al., 2018; Howells et al., 2019). None of the studies reported improvement or regression in the colon cancer.

## Discussion

Curcumin is the main constituent found in the Indian spice curcuma longa. It is the subject of research interest by investigators for use in various ailments, including cancer. Our motivation for this review is borne on the fact that the incidence of colon cancer in the Indian-subcontinent is among the lowest of in all cancer cases, possibly due to chemoprotective effect from curcumin and yet, there has yet to be a viable, regulatory approved curcumin medication on the market for use in treating colon cancer. There is a significant amount of research conducted at the experimental level and most appear to indicate the usefulness of curcumin *in vitro*.

Our objective was thus to systematic review the role of curcumin in colon cancer treatment. Curcumin was administered alone, in combination with other chemotherapeutics or through relevant formulation. Curcumin doses administered were as high as 3 g and was well tolerated in most of the subjects. However, the tumor regression was not reported in any of the study. It would seem that clinical effects of curcumin in managing colon cancer appears inconclusive.

Currently, the mainstay in the treatment of colon cancer relies mostly on 5-flourouracil and folinic acid (FOLFOX). Even though FOLFOX is relatively well-tolerated, dose-related adverse effects are often reported (Argyriou et al., 2013). Overcoming chemo-resistance and improving tolerability is the prime motivation in cancer research. In this regard, the use of dietary or plant-based agents are being recognized to offer potentially favorable toxicity profiles and likely to be better tolerated by patients than their chemotherapeutic cousins. Curcumin, derived from the Indian spice turmeric is arguably among the top contenders with wide therapeutic acclaim, including colon cancer. Worldwide clinical studies aimed at utilizing curcumin in treating colon cancer attest to the confidence amongst researchers in its therapeutic usefulness (Hsieh, 2001; Fang et al., 2014; Greil et al., 2018; Howells et al., 2019). In all of the studies presented in the current systematic review, curcumin was aptly, administered orally, with the exception of a few (Hsieh, 2001). Indeed, curcumin appears to be well-tolerated with doses of up to 3 mg/ kg as the allowable daily intake according to the WHO and other regulatory authorities (Kocaadam and Şanlier, 2017). Notwithstanding, the plasma levels of curcumin following oral administration is significantly low, largely attributable to its poor aqueous solubility. The maximum plasma concentration of curcumin following oral administration of up to 12 g of curcumin is less than160 nmol/l (Vareed et al., 2008). This poor solubility of curcumin in gastrointestinal media is compounded by sulphation and glucuronidation metabolic processes during traversing the gastrointestinal tract so that that the blood level is always bound to be low. In a study by Garcea et al. (Greil et al., 2018), lack of quantifiable blood concentration of curcumin in patients was found to be consistent in other clinical studies.

It is important to recognize that in the treatment of colon cancer via orally administered agents, systemic blood level is not consequential and it is more rational to achieve localized drug concentrations within the colon tissue or more appropriately, within the colon tumor (Hewlings and Kalman, 2017; Meng et al., 2018). Most of the clinical studies utilizing curcumin on patients involved the underlying condition being expressed at various stages. It is thus challenging to calibrate the efficacy of curcumin in such patients since drug resistance is associated with progression of the disease. Owing to its poor solubility, doses constituting orally administered pure curcumin are significantly higher than when used through formulation intervention. This has warranted the application of a variety of formulation interventions for the delivery of curcumin in clinical trials (Jin et al., 2009; Wang et al., 2019; Wong et al., 2019; Critical Appraisal tools, 2021). On the other hand, curcumin has also been delivered for clinical use in the unformulated pure form. Indeed, preclinical data appears to indicate that utilizing pure curcumin as an adjunct to established chemotherapeutics yields clinical dividends (Kocaadam and Şanlier, 2017). This seeming ‘interchangeability’ between formulated and pure curcumin is borne on the fact that putative efficacy of curcumin appears to be driven by several biochemical mechanisms, and mainly attributable to reduction in inflammation mediators, metastasis, increase in cell cycle arrest and apoptosis (Plummer et al., 1999; Irving et al., 2011; Park et al., 2013; James et al., 2015; Hewlings and Kalman, 2017; Meng et al., 2018). Increase in apoptosis has been observed when curcumin is used in this wise (Howells et al., 2007). Thus, phase I clinical trials based on diet-derived putative agents in patients is being advanced due to the fact that molecular targets are the same as those for chemotherapeutic agents.

In a controlled clinical trial involving curcumin and FOLFOX, authors contend that the treatment represent a safe and tolerable option (Schiborr et al., 2014). Colon cancer management through formulation intervention has customarily been in the form of micron or submicron delivery systems. In this regard, much of the pharmacokinetic data on curcumin formulations are promising. For example, a micellar formulation of curcumin resulted in an AUC of 12,147.7 nmol/ L. h whilst the micronisate formulation registered 4,547.5 nmol/ L. h, compared to only 65.6 nmol/ L. h from unformulated curcumin (Antony et al., 2008; Schiborr et al., 2014). In another study conducted on healthy volunteers, the AUC of curcumin encapsulated with turmeric essential oils was 8,690.0 nmol/ L. h (Howells et al., 2007). Furthermore, curcumin administered as phosphatidylcholine complex presented an AUC of 1765 nmol/ L. h (Cuomo et al., 2011). On the other hand, non-detectable blood level was reported in colon cancer patients administered unformulated curcumin, albeit the doses administered were much lower than those in healthy patients: 0.9 g (Sharma et al., 2004), and 1.8 g (Garcea et al., 2005), notwithstanding, non-detectable systemic concentrations were reported in these patients even at 2 g dose (Carroll et al., 2011). Oral consumption of up to 4 g of curcumin yields 10 nmol per Gram of colorectal mucosa. This level of colon tissue concentration, might be clinically relevant in the management of colon cancer, even if only trace levels are manifested systemically (Carroll et al., 2011). Thus, the clinical feasibility of utilizing curcumin in treating colon cancer remote from the gastrointestinal tract is questionable, unless some form of formulation intervention is applied. Research has also been directed toward modifying the physical properties of curcumin via chemical syntheses with the aim of enhancing its therapeutic use (Lin et al., 2006; Fang et al., 2014; Khor et al., 2019) Crucially, clinically relevant data can be discerned from *in vitro* studies conducted using some of these synthetic analogues of curcumin, but the roadmap to clinical application is still distant at the moment. The systematic review indicates that curcumin holds some clinical usefulness in treating colon cancer, albeit inconclusive. We also note the clinical advantage curcumin manifests in colon cancer treatment when administered through formulation intervention. Research scope between scientists working on synthetic derivatives of curcumin and formulation scientists seeking to present the most effect form of delivery of curcumin on one hand, and clinicians on the other has overlapped in recent years. The authors believe that the growth of this overlap is the key to unlocking the full clinical potential of curcumin not only in colon cancer but other clinical conditions as well.

## Conclusion

There is a huge volume of scientific investigations on the use of curcumin in therapy, however we are yet to traverse the threshold of clinically relevant benchmark. In the present systematic review, our focus was on colon cancer and the clinical usefulness of curcumin. The findings from this review indicate that curcumin in generally very well tolerated with doses in the range on 3 g. The side effects reported were common in all clinical trials, and mainly gastrointestinal. There was no regression in tumor size when curcumin was administered alone or in combination with other chemotherapeutics. Pharmacokinetic data indicate an almost 40-fold increase in blood levels in cases where curcumin was administered through formulation intervention compared administration in pure form. Until recently, there had been a gap between scientists and clinicians in harnessing the full potential of curcumin for clinical applications. This gap has narrowed in recent years as we see more formulations of curcumin being investigated *in vitro* and synthetic analogues of curcumin also being tested *in vitro*. These investigations along with clinical studies will need to auger in concert for the full clinical potential of curcumin to materialize.

## Data Availability

The original contributions presented in the study are included in the article/Supplementary Material, further inquiries can be directed to the corresponding author.
